# Luminescence complementation technology for the identification of MYC:TRRAP inhibitors

**DOI:** 10.18632/oncotarget.28078

**Published:** 2021-10-12

**Authors:** Edmond J. Feris, John W. Hinds, Michael D. Cole

**Affiliations:** ^1^Department of Molecular and Systems Biology, Geisel School of Medicine at Dartmouth College, Hanover, NH 03755, USA; ^2^Norris Cotton Cancer Center, Dartmouth-Hitchcock Medical Center, Lebanon, NH 03756, USA

**Keywords:** cancer, drug discovery, targeted therapy, oncogene, transcription factor

## Abstract

Mechanism-based targeted therapies have exhibited remarkable success in treating otherwise untreatable or unresectable cancers. Novel targeted therapies that correct dysregulated transcriptional programs in cancer are an unmet medical need. The transcription factor MYC is the most frequently amplified gene in human cancer and is overexpressed because of mutations in an array of oncogenic signaling pathways. The fact that many cancer cells cannot survive without MYC – a phenomenon termed “MYC addiction” – provides a compelling case for the development of MYC-specific targeted therapies. We propose a new strategy to inhibit MYC function by disrupting its essential interaction with TRRAP using small molecules. To achieve our goal, we developed a platform using luminescence complementation for identifying small molecules as inhibitors of the MYC:TRRAP interaction. Here we present validation of this assay by measuring the disruption of TRRAP binding caused by substitutions to the invariant and essential MYC homology 2 region of MYC.

## INTRODUCTION

Recent years have been marked by a focused recalibration of drug discovery engines in the pharmaceutical and biotechnology worlds. Rising costs of research and development hinder the pursuit of targets without the greatest substantial value. Historical failure coupled with low probability of success and lack of novel approaches have focused efforts into the pursue of new therapeutic strategies. However, mechanism-based targeted therapies have exhibited remarkable success in treating otherwise untreatable or unresectable cancers [1]. Amongst these, the most successful are chemical or biological entities that specifically target cancerous cellular states and have minimal effects on normal cellular programs.

Novel targeted therapies that correct dysregulated transcriptional programs in cancer are an unmet medical need [2]. The transcription factor MYC is the most frequently amplified gene in human cancer and is overexpressed because of mutations in an array of oncogenic signaling pathways [3, 4]. The fact that many cancer cells cannot survive without MYC – a phenomenon termed “MYC addiction” – provides a compelling case for the development of MYC-specific targeted therapies [2, 5–8]. Therefore, MYC has been recognized as the ‘most wanted’ target in cancer for decades, but most efforts have met with inescapable off-target toxicity [9–11]. Despite attempts at inhibiting MYC expression, its direct interaction with DNA and its obligate partner MAX, or any indirect MYC effectors, no clinically useful drug has emerged in nearly 20 years due to poor accessibility and specificity [9, 11, 12].

Since MYC has no inherent enzymatic activity, it has been inaccurately described as an “undruggable” target [9–11]. However, it does have a functional DNA-binding domain and a transactivation domain (TAD). The DNA-binding domain requires a protein-protein interaction (PPI) with its obligate partner MAX. Several labs have attempted to find small molecules that inhibit MYC:MAX with limited success [9–11]. It is informative to point out the timeline of MYC:MAX inhibitor research. The first MYC:MAX inhibitor was reported in 2002, and it functioned at 50–100 μM [13]. Improved analogs of this inhibitor (~25 μM) were reported in 2007 [14] and other inhibitors have been reported more recently [12, 15–18]. However, the specificity of these inhibitors for MYC:MAX is often unclear.

We propose a new strategy to inhibit MYC function by disrupting the MYC:TRRAP interaction using small molecules. TRRAP is one of the best characterized MYC cofactors and is an essential component of various histone acetyltransferase (HAT) complexes [5, 19–23]. The identification of TRRAP as an essential MYC cofactor established a link to HAT complexes containing GCN5 and TIP60 and provided an important mechanistic insight into MYC function [10, 19, 21–23]. The MYC:TRRAP interaction occurs at a precise motif in the TAD of MYC known as the MYC Homology Box 2 (MB2) [5, 21]. The importance of MB2 in MYC-driven tumorigenesis is well established in cellular assays and animal models [24, 25], presumably because it is necessary for the MYC:TRRAP interaction. Therefore, the TAD of MYC, MB2, and MYC:TRRAP are required for MYC-driven transactivation and cancer promotion. Furthermore, MB2 is unique and nearly invariant in evolution [5, 19], suggesting that it is an ideal site for inhibiting MYC function.

Here we present a robust and high-throughput-amenable platform that can be used to identify inhibitors of the MYC:TRRAP interaction. Using luminescence complementation and the minimal MYC and TRRAP interacting domains, we developed a PPI assay that results in an active luciferase enzyme when MYC:TRRAP complexes form. We used the NanoLuc^®^ Binary Technology (NanoBiT^®^), a split version of the NanoLuc^®^ luciferase (a 19.1 kDa protein that produces an ATP-independent glow-type luminescence with half-life > 2 h; Promega Corporation) intended for measurement of PPIs in live cells [26]. Unlike co-IPs and other binding assays, the NanoBiT^®^ system enables quantifiable measurements without cell lysis. With high sensitivity and broad dynamic range, bioluminescent methods have proven useful for many applications, including binding assays and drug discovery. We validated our assay by identifying substitution mutations in MB2 that result in loss of TRRAP binding and correlate with loss of MYC-driven cellular transformation.

## RESULTS

### TRRAP 2033-2283 is the minimal MYC-binding domain

**Figure 1:**
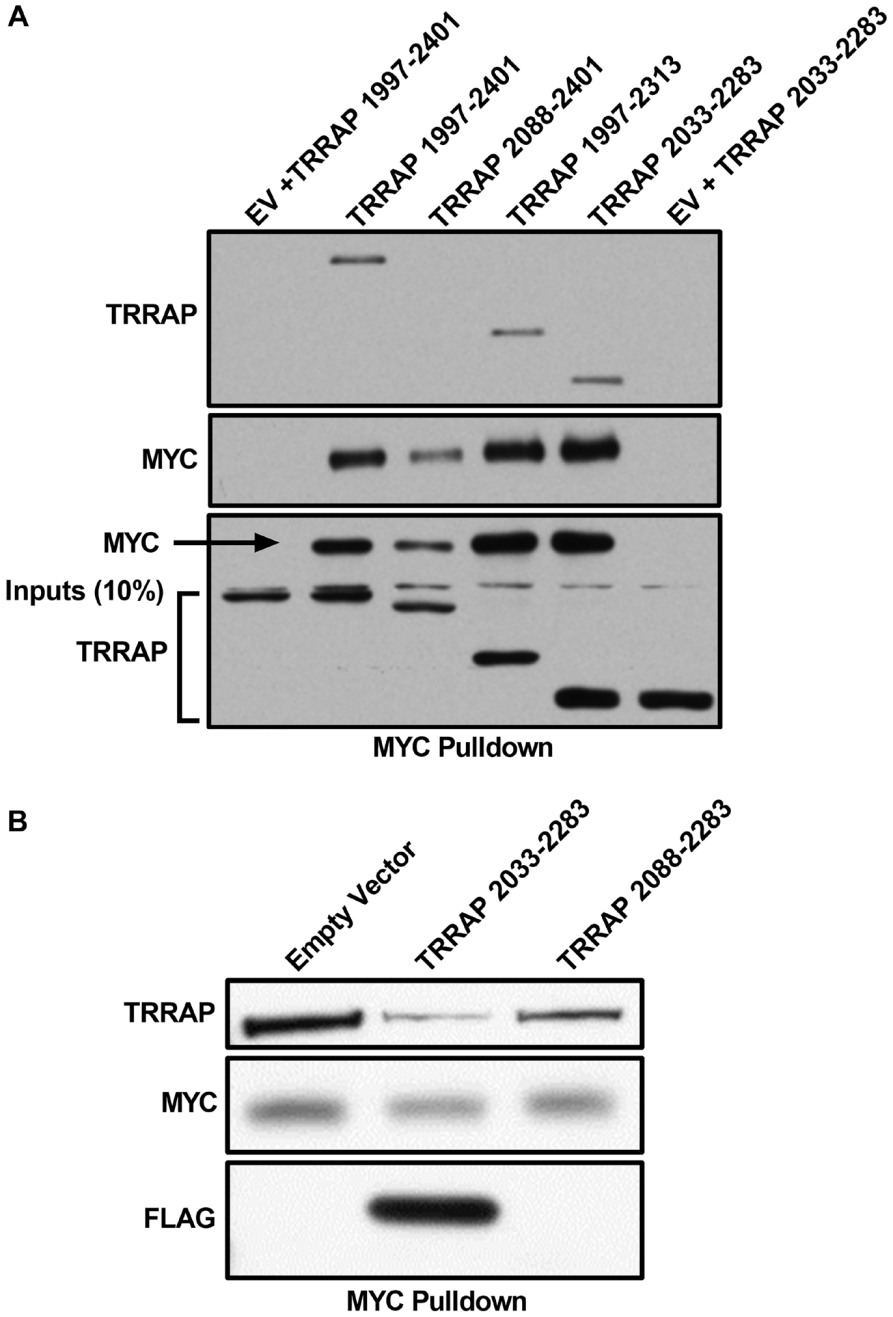
TRRAP 2033-2283 is the minimal MYC-binding domain. (**A**) Western blot analysis of a co-immunoprecipitation experiment where the indicated regions of TRRAP were cloned into a CMV-FLAG expression vector and transfected into HEK293T cells. Proteins were co-expressed with PYO-tagged full-length MYC and then MYC was IP’d with anti-PYO beads. Co-IP was assessed by western blot with anti-FLAG. The most critical binding domain is within residues 2033-2088, consistent with previous results [5], and the minimal domain TRRAP 2033-2283 is established. (**B**) Western blot analysis of a co-immunoprecipitation experiment where the indicated regions of TRRAP were cloned into a CMV-FLAG expression vector and transfected into HEK293T cells. Endogenous MYC was IP’d with C-33 beads (Santa Cruz Biotechnology). The TRRAP and FLAG blots show TRRAP 2033-2283 can compete off the endogenous native MYC:TRRAP complex in cells, but the same domain of TRRAP lacking the critical region 2033-2088 does not show the same level of competition.

We previously showed that MYC 1-190 is sufficient for a stable MYC:TRRAP interaction [5], and we wanted to identify the minimal domain of TRRAP that is sufficient for complex formation. TRRAP 1997–2401 binds to MYC and TRRAP 1997–2088 is required but not sufficient to sustain the MYC:TRRAP interaction in co-IP assays [5]. Further deletion studies show that a minimal domain of TRRAP (2033–2283) is sufficient for protein complex formation in co-IP experiments (Figure 1A). To validate this mapping, co-IP experiments were performed to test if TRRAP 2033–2283 is essential in the native MYC:TRRAP complex. A competition experiment between the TRRAP 2033–2283 domain and native MYC:TRRAP complexes shows that overexpression of the critical TRRAP domain inhibited formation of native MYC:TRRAP complexes (Figure 1B). This effect was attenuated when the core internal binding region of TRRAP (2033–2088) was not present (Figure 1B, lane 3).

### MYC:TRRAP luminescence complementation platform is MB2-dependent

**Figure 2:**
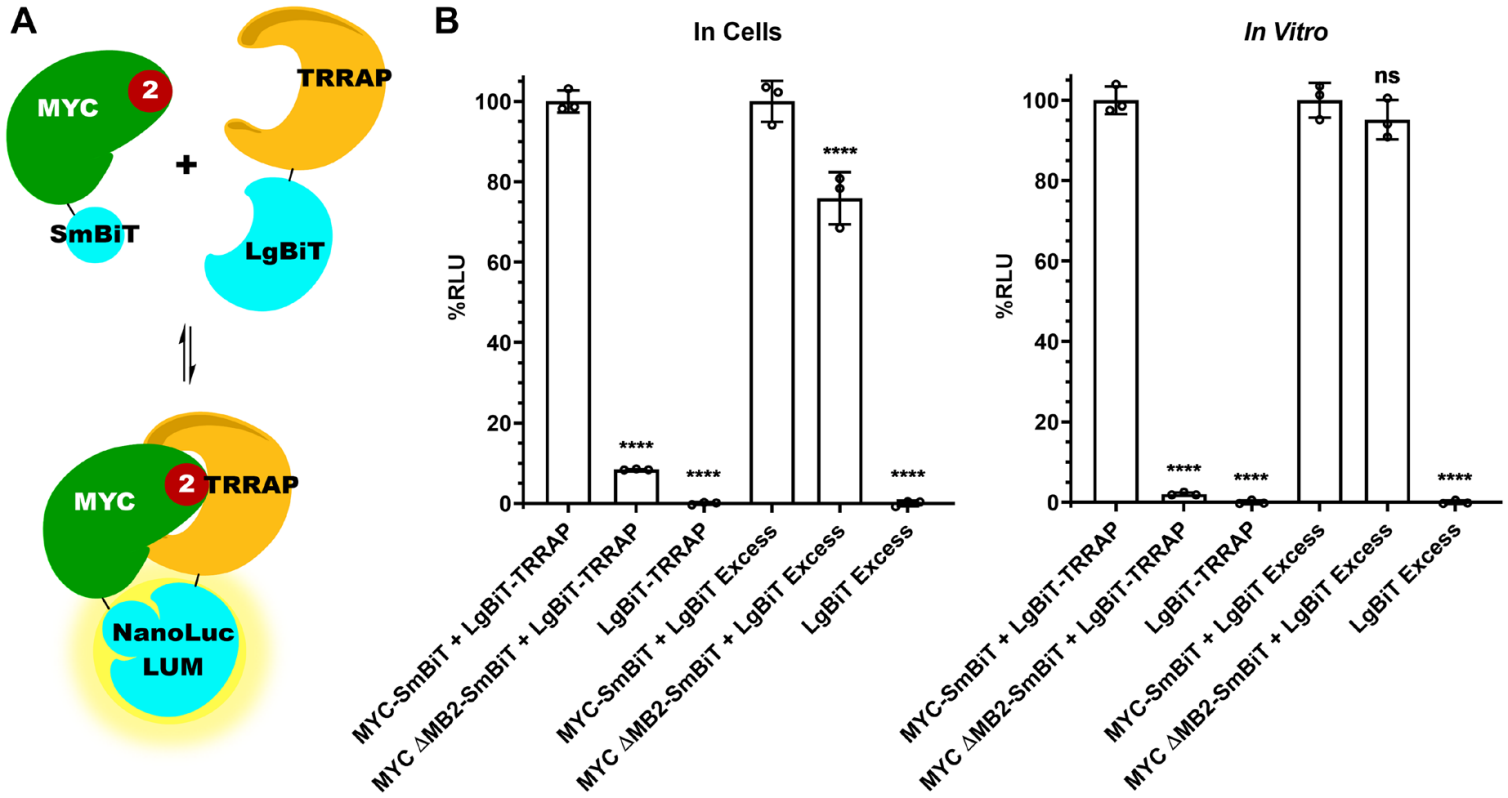
MYC:TRRAP luminescence complementation platform is MB2-dependent. (**A**) Schematic representation of MYC 1-190 and TRRAP 2033-2283 fused to each subunit of the NanoLuc^®^ Binary Technology (NanoBiT) system. (**B**) Luminescence measurements of cells transfected with TRRAP 2033-2283-LgBiT and SmBiT-MYC 1-190 with and without MB2 and alone. The LgBiT subunit was co-transfected in excess with SmBiT-MYC 1-190 (+/− MB2) and alone. The panel on the left shows measurements carried out in cells while the right panel shows measurements in cellular extracts. A 10-fold decrease is observed with the deletion of MB2. MYC binding to TRRAP is MB2 dependent, validating *in vivo* immunoprecipitation experiments with native proteins [5,21]. An unpaired Student’s *t*-test was performed to determine standard deviation and statistical significance. *P*-value ≤ 0.05 was considered statistically significant. Error bars represent SD and ns: *p* > 0.05, ^*^: *p* ≤ 0.05, ^**^: *p* ≤ 0.01, ^***^: *p* ≤ 0.001, ^****^: *p* ≤ 0.0001.

Defining the minimal domains that form the MYC:TRRAP complex allowed us to develop an assay for PPI using the NanoBiT^®^ PPI system, which fuses the Large Binary Technology (LgBiT) and Small Binary Technology (SmBiT) subunits to different interacting proteins. Briefly, residues 2033–2283 of TRRAP and 1-190 of MYC were respectively cloned into CMV-driven vectors containing the LgBiT (18kDa) and SmBiT (11aa) subunits to monitor the MYC:TRRAP PPI (Table 1). Upon protein dimerization of MYC:TRRAP, the NanoBiT^®^ subunits complement and form a highly active luciferase enzyme (Figure 2A).

**Table 1:** Key resources table

Reagent or Resource	Source	Identifier
Antibodies		
Rabbit polyclonal anti-MYC	Santa Cruz Biotechnology	sc-764
Rabbit polyclonal anti-FLAG	Millipore Sigma	F7425
Mouse monoclonal anti-Glu-Glu Epitope Tag Affinity Matrix	BioLegend	AFC-115P-1000
Mouse monoclonal anti-MYC agarose conjugate	Santa Cruz Biotechnology	sc-42 AC
Rabbit polyclonal anti-MYC	Cell Signaling Technology	9402
Rabbit polyclonal anti-TRRAP	Bethyl Laboratories	A301-132A
Mouse monoclonal anti-MAX	Santa Cruz Biotechnology	sc-8011
Mouse monoclonal anti-V5	Invitrogen	46-0705
Mouse monoclonal anti-Vinculin	Santa Cruz Biotechnology	sc-73614
Critical Commercial Assays		
NanoBiT^®^ PPI System	Promega	N2014
Nano-Glo^®^ Live Cell Assay System	Promega	N2012
ExpiFectamine™ 293 Transfection Kit	Gibco™	A14524
LipoD293™	SignaGen	SL100668
Experimental Models: Cell Lines		
HEK293T	ATCC	CRL-3216, RRID:CVCL 0063
Expi293F	Gibco™	A14527, RRID:CVCL D615
MCF-10A	ATCC	CRL-10317, RRID:CVCL 0598
Recombinant DNA		
Plasmid: CβP MYC 1-190	This paper	N/A
Plasmid: CβF TRRAP 2033-2283	This paper	N/A
Plasmid: Cβ2N MYC 1-190	This paper	N/A
Plasmid: Cβ1C TRRAP 2033-2283	This paper	N/A
Plasmid: pCDH V5-MYC 1-439	This paper	N/A
Software and Algorithms		
Prism	GraphPad	N/A
Image Studio™	LI-COR	N/A

Next, we measured MYC:TRRAP complex formation using luminescence complementation. The minimal interacting domains described above gave a robust luminescence, whereas deletion of MYC Homology Box 2 (MB2, amino acids 128-144) reduced luminescence by 90% both in cells and in cellular extracts (Figure 2B compare bars 1 and 2). Thus, this luciferase assay appears to recapitulate the *in vivo* MYC:TRRAP interaction [5, 21]. As a control, we measured the expression of both MYC and MYC ΔMB2 using the NanoBiT system. The LgBiT and SmBiT subunits are able to associate independently with a Kd of 190 μM [27]. Therefore the LgBiT subunit alone was co-transfected in excess to allow independent association with the SmBiT subunit in both MYC and MYCΔMB2. This provides a measurement that shows equal expression for both MYC and MYCΔMB2 constructs (Figure 2B lanes 4 and 5).

One additional LgBiT-TRRAP construct was tested for luminescence. To confirm the importance of the TRRAP 2033-2088 domain, luminescence was measured with TRRAP 2033-2283 compared to a similar construct lacking the MYC binding region, TRRAP 2088-2283 (Supplementary Figure 1). Like MB2, the absence of TRRAP 2033-2088 diminishes binding, consistent with co-IP experiments [5].

### MB2 substitutions affect TRRAP binding and cellular transformation

**Figure 3:**
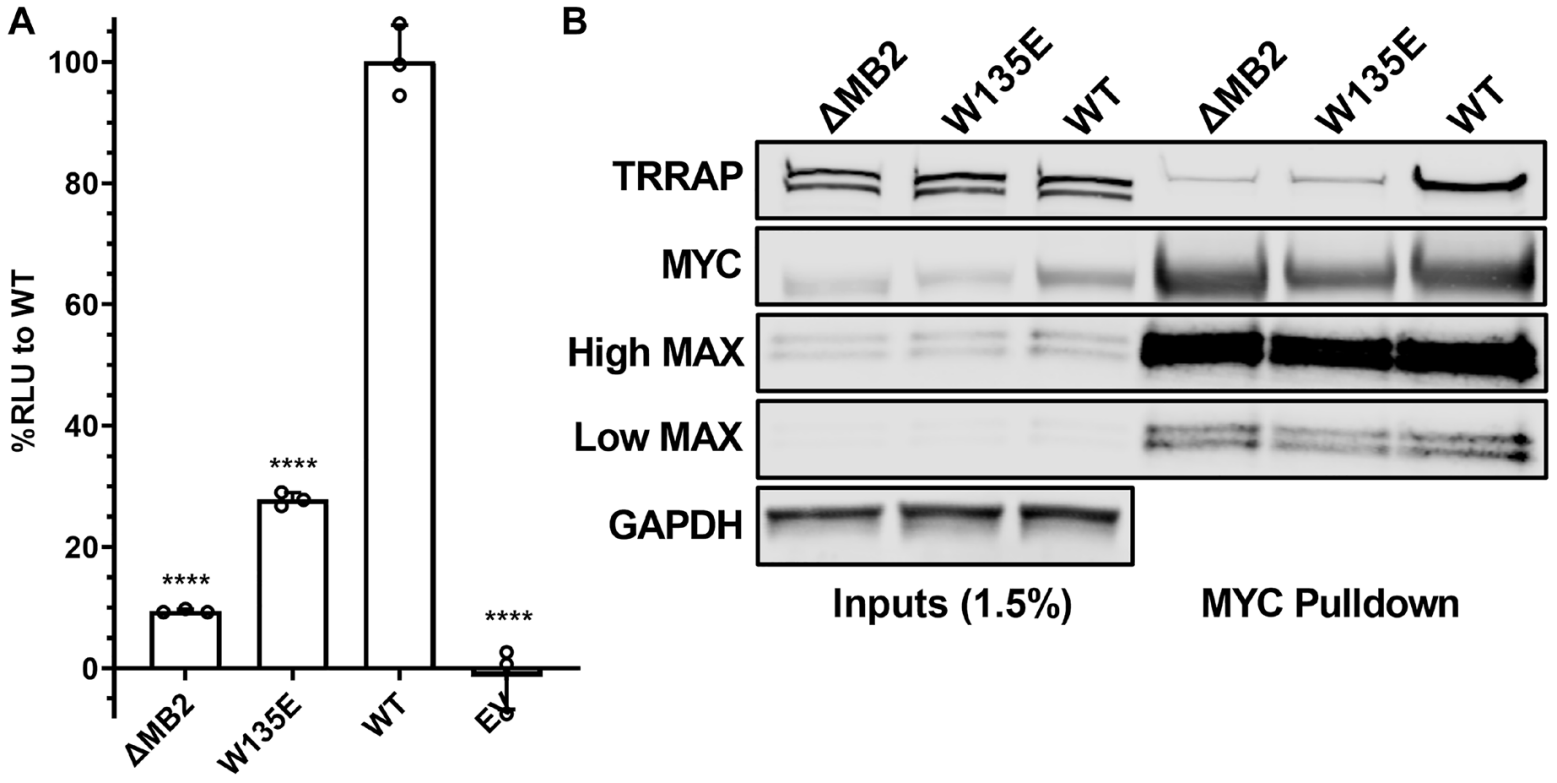
W135 is essential for MYC:TRRAP. (**A**) Luminescence measurements of cells transfected with TRRAP 2033-2283-LgBiT and empty vector, SmBiT-MYC 1-190 (+/− MB2), and with a W135E substitution. These results indicate the importance of W135 for the MYC:TRRAP interaction. An unpaired Student’s *t*-test was performed to determine standard deviation and statistical significance. *P*-value ≤ 0.05 was considered statistically significant. Error bars represent SD and ns: *p* > 0.05, ^*^ *p* ≤ 0.05, ^**^ *p* ≤ 0.01, ^***^ *p* ≤ 0.001, ^****^ *p* ≤ 0.0001. (**B**) Western blot analysis of a coimmunoprecipitation experiment where the native TRRAP and MYC proteins or the indicated MYC mutants were co-transfected into Expi293F cells. PYO-tagged full-length MYC was co-expressed with FLAG-tagged full-length TRRAP and then MYC was IP’d with anti-PYO beads. High/low MAX refers to exposure time. These results validate the MYC:TRRAP NanoBiT platform and indicate that it can provide quantifiable measurements that recapitulate the binding interaction of the native MYC:TRRAP complex.

To validate luminescence complementation measurements further, we tested if this assay could provide reliable measurements of MYC:TRRAP binding perturbation caused by a single residue substitution mutation (W135E). MYC W135 is within the core of MB2, and previous reports showed that W135 is essential for MYC-driven cellular transformation and transactivation [24, 28, 29]. Nevertheless, the involvement of W135 in TRRAP binding has been unclear. Using the luminescence complementation platform, we found that a W135E substitution reduced MYC:TRRAP binding in cells to ~25% of WT (Figure 3A). We also validated this measurement using the native MYC:TRRAP complex in a co-IP experiment (Figure 3B).

**Figure 4:**
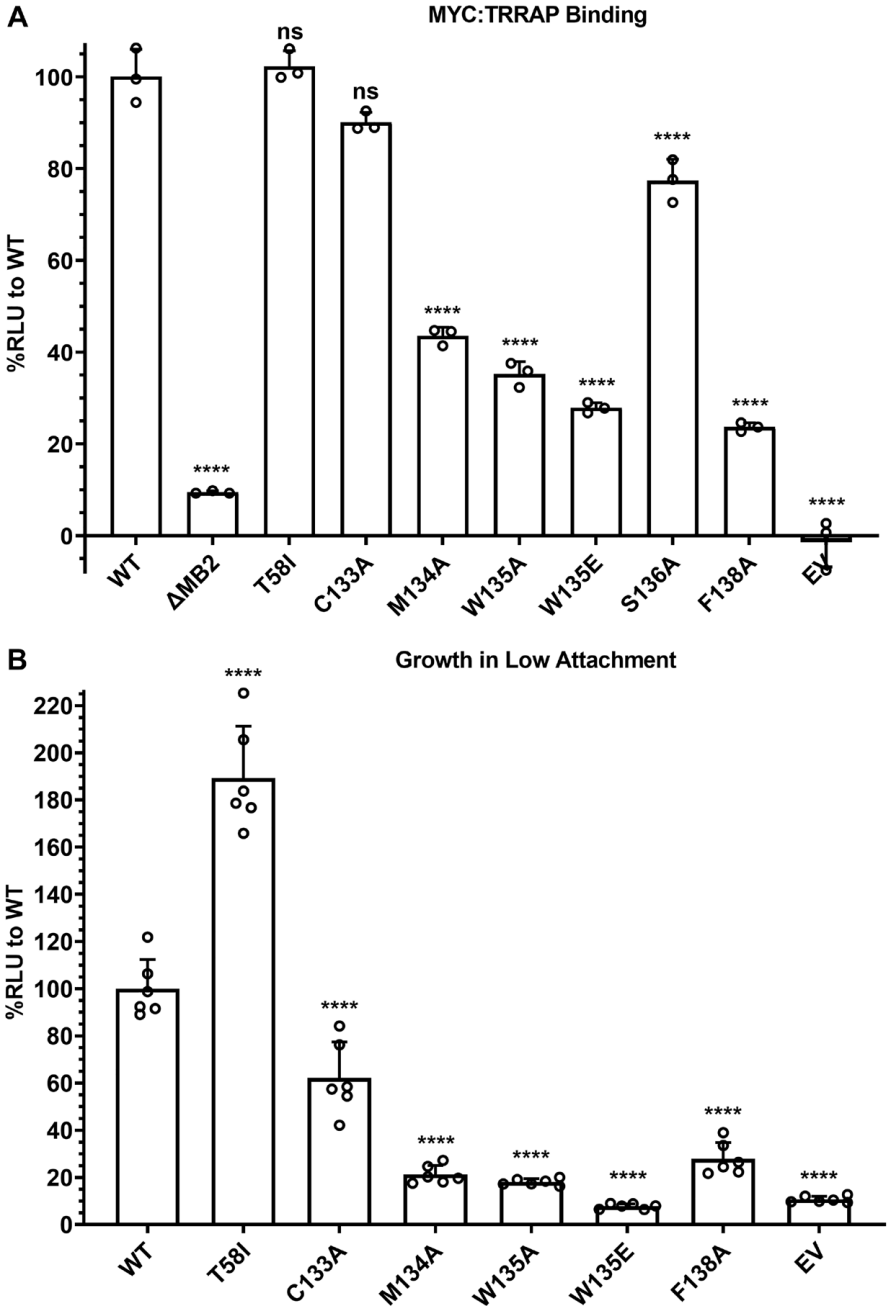
MB2 substitutions affect TRRAP binding and cellular transformation. (**A**) Luminescence measurements of cells transfected with TRRAP 2033-2283-LgBiT and the indicated SmBiT-MYC 1-190 or mutants. These results show the effects of substitution mutations in MB2 on TRRAP binding. An unpaired Student’s *t*-test was performed to determine standard deviation and statistical significance. *P*-value ≤ 0.05 was considered statistically significant. Error bars represent SD and ns: *p* > 0.05, ^*^ *p* ≤ 0.05, ^**^ *p* ≤ 0.01, ^***^ *p* ≤ 0.001, ^****^ *p* ≤ 0.0001. (**B**) Luminescence measurements indicating proliferation of MCF-10A cells evaluated for their ability to grow as spheroids on ultra-low adherent plates after 7 days. The indicated MYC mutants were constitutively overexpressed exogenously. These results indicate which substitution mutations in MB2 are disruptive of MYC-driven cellular transformation. An unpaired Student’s *t*-test was performed to determine standard deviation and statistical significance. *P*-value ≤ 0.05 was considered statistically significant. Error bars represent SD and ns: *p* > 0.05, ^*^ *p* ≤ 0.05, ^**^ *p* ≤ 0.01, ^***^ *p* ≤ 0.001, ^****^ *p* ≤ 0.0001.

To further explore the MB2-dependence of MYC:TRRAP binding, a series of point mutations was created in SmBiT-MYC 1-190, and any changes in TRRAP 2033-2283 binding were measured via luminescence complementation. Residues with high evolutionary conservation (M134, W135, S136, and F138) were each substituted with alanine residues [30]. We also included the most common MYC mutation in cancer, T58I/A/P/N [31–38]. Unsurprisingly this cancer-associated mutation appeared to increase TRRAP binding. However, after normalization for MYC expression there was no apparent effect on TRRAP binding. The T58I mutation has been reported as a MYC protein stabilizing mutation. The higher MYC expression results in more MYC:TRRAP complexes but there is no increased binding to TRRAP at comparable MYC expression levels. Therefore, the expression level for each construct had to be determined with the previously described luminescence-based assay (Supplementary Figure 2) and used to normalize TRRAP binding measurements (Figure 4A). Alanine substitutions at the most conserved MB2 residues (M134A, W135A, S136A, F138A) confirmed their importance in the MYC:TRRAP interaction, with decreased binding to ~20–40% of WT (Figure 4A). Since both W135A and W135E are defective, it is likely that the bulkier hydrophobicity of the tryptophan is critical. M134A also caused a significant decrease in luminescence complementation, though not as much as W135A/E. Since F138A showed the same decrease in luminescence as W135A/E, it suggests that F138 may have an important participation in the MYC:TRRAP interaction. In contrast, the most common and recurrent MYC mutation in cancer, T58I, showed no change in TRRAP binding, despite a significant increase in expression (Supplementary Figure 2).

To test if TRRAP binding measurements with MB2 substitution mutants affect MYC activity, we tested if these mutations have a correlation with disruption of MYC-driven cellular transformation as measured by anchorage independent growth (Figure 4B). MCF-10A cells overexpressing MYC have been proposed to be a cellular model for triple negative breast cancer [25, 39, 40]. A Growth in Low Attachment (GILA) assay was developed as a quantifiable measure of anchorage-independent growth which is a marker of cellular transformation [41]. GILA measurements were performed with MCF-10A cells engineered with ectopic expression of full-length WT MYC or with the indicated substitutions (Figure 4B, Supplementary Figure 3). The results show that each of these residues is important for MYC function which correlates with the MYC:TRRAP interaction assay.

## DISCUSSION

Cancer vulnerabilities that can be targeted without harming normal cells have led to major breakthroughs in the development of new treatment options [1]. Studying molecular events that provide cancer cells with proliferative advantages is at the center of these endeavors. The MYC:TRRAP interaction is a MYC vulnerability in cancer and presents an opportunity for drug discovery. Here we show a platform that can be used to identify small molecules as inhibitors of the MYC:TRRAP interaction. Inhibition of MYC and TRRAP binding caused by substitution mutations in MB2 was tested measuring luminescence using a PPI complementation assay, both in cells and in cellular extracts. A decrease in luminescence complementation is indicative of a residue that may participate directly in contacts between MYC and TRRAP. The assay was further corroborated by co-IP measurements of the native protein complex and by a MYC-driven cellular transformation assay.

In contrast, achieving MYC inhibition is possible by targeting the DNA-binding domain, but most efforts have met with inescapable off-target toxicity [10, 11]. However, inhibiting MYC with a compound that binds or targets MB2 and its interaction with TRRAP can be a more fruitful strategy [5, 19]. The fact that single amino acid substitutions in MB2 were able to abolish the MYC:TRRAP interaction provides convincing evidence that the interaction relies on a sensitive pocket that can be disrupted by small molecules, rather than a large interface like the one found in the MYC:MAX interaction. Inhibition efforts directed at a smaller defined pocket, such as MB2, would likely result in more specific inhibitors. Higher specificity is an advantage over the MYC:MAX inhibitors to date because it allows for precise titrations to achieve lower toxicity. This function identifies an appropriate therapeutic window where only cancer cells are vulnerable to the downregulation of MYC activity. Compounds identified with this platform can lead to a new generation of drugs targeting this unique region of the MYC protein. The uniqueness of MB2 will likely result in very specific therapeutic compounds with minimal off-target effects, and its essentiality assures that tumor cells cannot escape a treatment strategy that targets MYC’s MB2 in cancer. Furthermore, the finding that individual hydrophobic residues within MB2 are essential for the MYC:TRRAP interaction and MYC-driven cellular transformation can be exploited to guide a drug discovery effort. For example, targeted sets of screening compound libraries could be prioritized given that the MYC:TRRAP interaction appears to be driven primarily by hydrophobic interactions. Additionally, our MYC:TRRAP NanoBiT assay could be altered to enhance hydrophobic interactions and raise the potency of the interaction and any potential new inhibitors.

Additionally, it is worth addressing a novel aspect of the biology and importance of the MYC:TRRAP interaction. The structure of Tra1p (TRRAP yeast homolog) has been described alone and in complex with part of NuA4 [42, 43]. Tra1p is composed of α-helical solenoid repeats, spanning both HEAT and FAT domains, which account for 86% of its mass. For TRRAP, the site of interaction with MYC in the HEAT domain is a predicted intrinsically disordered region (IDR; 2033-2088). Not only does this region contain the two major phosphorylation sites in TRRAP, but it also contains a nuclear localization signal [44–46]. As is the case for DNA-PKcs, it is possible that the IDR, as part of TRRAP’s HEAT domain, is involved in an allosteric mechanism of modulation of TRRAP-containing HAT complexes. MYC binding might cause conformational changes to these complexes that regulate their function. The IDR of TRRAP can serve as a ‘hot-spot’ sensor for cellular events that affect TRRAP and therefore HAT activity.

GCN5 and Tip60 have been reported to acetylate histone tails *in vitro* but cannot do so for an assembled nucleosome. Both enzymes require other members of the STAGA and NuA4 complexes respectively (TRRAP specifically) for efficient acetylation of assembled nucleosomes [47, 48]. Given these observations and TRRAP’s HEAT domain structural similarities with that of DNA-PKcs, TRRAP could be required for efficient HAT activity because it enables the presentation of lysine tails by denaturing nucleosomes. Its HEAT domain could help stabilize relaxed DNA within its large solvent-accessible channels. This model provides a rationale for TRRAP essentiality in MYC cancer biology.

## MATERIALS AND METHODS

### Resource availability

#### Lead contact

Further information and requests for resources and reagents should be directed to and will be fulfilled by Michael D. Cole.

#### Materials availability

Plasmids and cell lines generated in this study are available upon request unless there is a conflict of interest. This study did not generate any other new unique reagents.

#### Data and code availability

This study did not generate any large datasets or analysis code.

### Experimental model and subject details

*HEK293T* cell line (ATCC Cat# CRL-3216, RRID:CVCL_0063): maintained in DMEM (Corning^®^) supplemented with 10% fetal bovine serum (Corning^®^), 1% Penicillin-Streptomycin (Corning^®^), and prophylactic Plasmocin™ (InvivoGen) at 37°C with 5% CO_2_ and ≥ 80% relative humidity.

*Expi293F* cell line (Gibco™ Cat# A14527, RRID:CVCL_D615): maintained in Expi293™ Expression Medium (Gibco™) at 37°C with 8% CO_2_ and ≥ 80% relative humidity and 125 rpm shaking.

*MCF-10A* cell line (ATCC Cat# CRL-10317, RRID:CVCL_0598): maintained in DMEM/F12 (Corning^®^) supplemented with 5% horse serum (Invitrogen), 20 ng/mL EGF, 0.5 μg/mL Hydrocortisone, 100 ng/mL Cholera toxin, 10 μg/mL Insulin, 1% Penicillin-Streptomycin (Corning^®^), and prophylactic Plasmocin™ (InvivoGen) at 37°C with 5% CO_2_ and ≥ 80% relative humidity.

### Method details

#### Co-transfection

##### HEK293T

HEK293T cells were co-transfected with equal amounts of each plasmid using LipoD293™ *In Vitro* DNA Transfection Reagent per protocol (SignaGen). Cells were plated subconfluently 16–20 hours prior to transfection. After 24 hours, cells were lysed in F-buffer (10 mM TRIS pH 7.5, 50 mM NaCl, 30 mM sodium pyrophosphate, 5 mM ZnCl_2_, 10% glycerol, 50 mM NaF) containing 1% Triton-X and supplemented with 1 mM PMSF, 10 μM Leupeptin, 10 μM Pepstatin-A, and 10 μg/mL Aprotinin for co-immunoprecipitations.

##### Expi293F

Expi293F cells were co-transfected with adjusted ratios of each plasmid appropriate to the expression of its containing construct using ExpiFectamine™ 293 Transfection Kit per protocol (Gibco™). New plasmid preparations required optimization of the adjusted DNA ratios. Cells were transfected in flasks in batches of various volumes at 3 × 10^6^ cells/mL. A bluescript KS+ plasmid (Addgene) was used as carrier DNA when needed, and a pcDNA3.1 EGFP plasmid (ThermoFisher) was used as a fluorescence reporter to determine transfection efficiency. After 48 hours, luminescence measurements were taken or cells were lysed in F-buffer containing 100 μg/mL Digitonin supplemented with 1 mM PMSF, 10 μM Leupeptin, 10 μM Pepstatin-A, and 10 μg/mL Aprotinin.

##### Co-immunoprecipitation

Immunoprecipitations were performed using anti-PYO (Covance), or anti-MYC (C33 Santa Cruz Biotechnology) agarose pre-conjugated beads at 4°C for 16–20 h. Co-immunoprecipitation was analyzed by western blots with the following antibodies: MYC (sc-764 Santa Cruz Biotechnology) or MYC (9402 Cell Signaling Technology), TRRAP (A301-132A Bethel Laboratories), MAX (sc-8011 Santa Cruz Biotechnology), and FLAG (F7425 Millipore Sigma).

### Luminescence complementation

#### In cells

Transfected Expi293F cells were plated on 96-well white plates with clear bottoms (Greiner). White light-reflecting film (USA Scientific) was used to cover the bottom of the plates for luminescence measurements. Black light-absorbing film was used to cover the top of the plates for fluorescence measurements. All measurements were taken on a SpectraMax i3 instrument (Molecular Devices).

#### In vitro

Cells were lysed in the same volume of lysis buffer than that in which they were cultured. Protein concentration of cellular extracts were normalized using a standard BSA protein assay (Bio-Rad Laboratories). After lysis, cellular extracts were plated on 96-well white plates with clear bottoms (Greiner). White light-reflecting film (USA Scientific) was used to cover the bottom of the plates for luminescence measurements. Black light-absorbing film was used to cover the top of the plates for fluorescence measurements. All measurements were taken on a SpectraMax i3 instrument (Molecular Devices).

#### MCF-10A growth in low attachment assay (GILA)

MCF-10A cells were engineered with constitutive MYC overexpression or the indicated mutants using a pCDH EF1α-driven lentiviral vector with a puromycin resistance selectable marker. The protocol for GILA measurements was adapted from [41]. Briefly, after selection, 100 μL of cells were seeded at 10^4^ cells/mL in ultra-low attachment 96-well plates from Corning^®^. After a 7-day incubation, cell proliferation was quantified using CellTiter-Glo^®^ 2.0 Cell Viability Assay according to manufacturer’s instructions. All measurements were taken on a SpectraMax i3 instrument (Molecular Devices).

### Quantification and statistical analysis

All statistical analysis was performed in GraphPad Prism. Measurements were performed in triplicate at least. An unpaired Student’s *t*-test was performed to determine standard deviation and statistical significance. *P*-value ≤ 0.05 was considered statistically significant. Error bars represent SD and ns: *p* > 0.05, ^*^ *p* ≤ 0.05, ^**^ *p* ≤ 0.01, ^***^ *p* ≤ 0.001, ^****^ *p* ≤ 0.0001.

## SUPPLEMENTARY MATERIALS


