# Supplementary material for: Luminescence complementation technology for the identification of MYC:TRRAP inhibitors
Source: Oncotarget. 2021 Oct 12;12(21):2147–57. doi: 10.18632/oncotarget.28078 (PMC8522838; doi:10.18632/oncotarget.28078)
Supplement: Supplementary file 1 [file oncotarget-12-2147-s001.pdf]

Luminescence complementation technology for the identification of MYC:TRRAP inhibitors

SUPPLEMENTARY MATERIALS

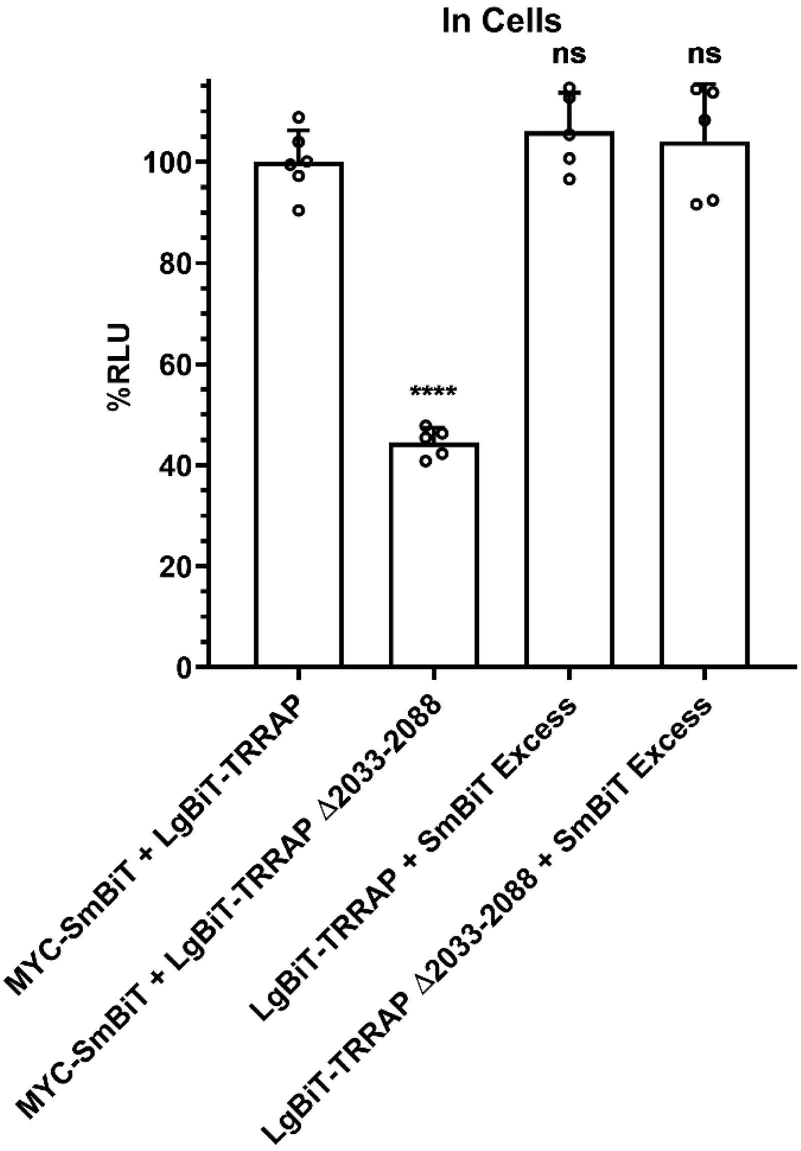

Supplementary Figure 1: TRRAP depends on 2033-2088 for MYC binding in cells. Luminescence measurements of cells transfected with the indicated MYC 1-190 and TRRAP 2033-2283 pairs or with SmBiT in excess. The graph shows TRRAP 2033-2283's dependence on 2033–2088 for MYC binding and equal expression of TRRAP 2033-2283 and TRRAP 2088-2283. An unpaired Student's *t*-test was performed to determine standard deviation and statistical significance. *P*-value ≤ 0.05 was considered statistically significant. Error bars represent SD and ns: $p > 0.05$, $^{*}p \leq 0.05$, $^{**}p \leq 0.01$, $^{***}p \leq 0.001$, $^{****}p \leq 0.0001$.

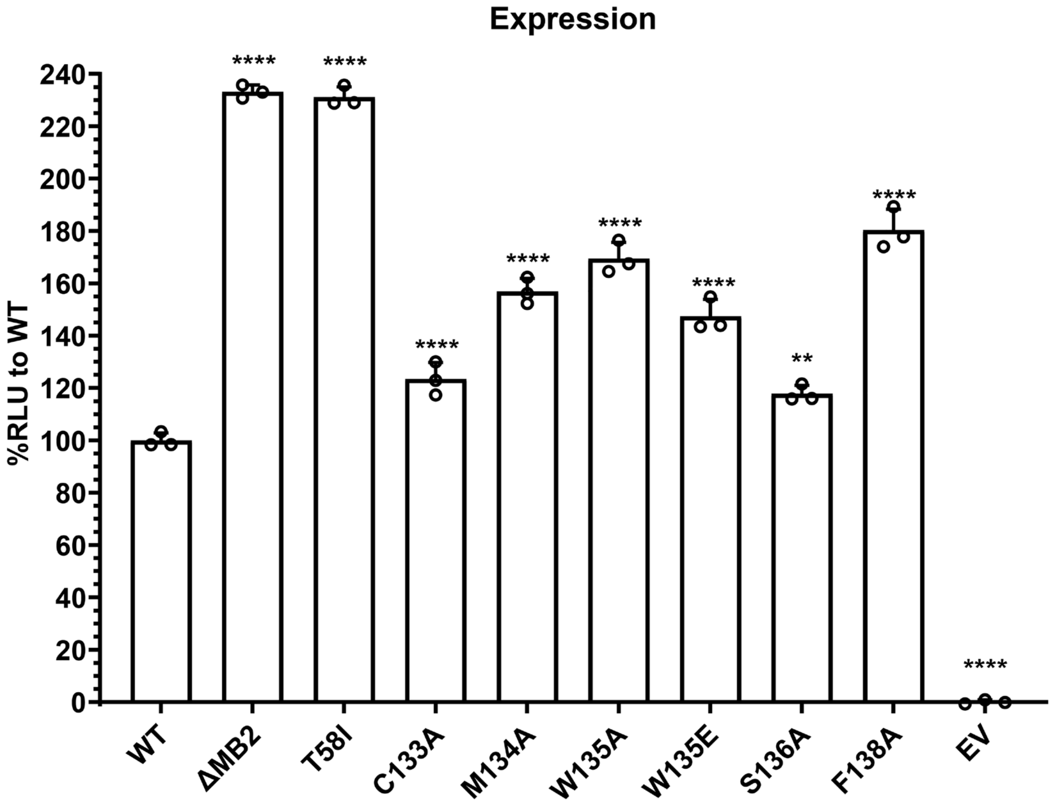

Supplementary Figure 2: Expression changes with MYC substitution mutations in MB2. Luminescence measurements of cells transfected with the LgBiT subunit in excess and the indicated MYC 1-190 or mutant pairs. The graph confirms that the T58I mutation stabilizes MYC expression in cells as previously reported. An unpaired Student's *t*-test was performed to determine standard deviation and statistical significance. *P*-value ≤ 0.05 was considered statistically significant. Error bars represent SD and ns: $p > 0.05$, $^{*}p \leq 0.05$, $^{**}p \leq 0.01$, $^{***}p \leq 0.001$, $^{****}p \leq 0.0001$.

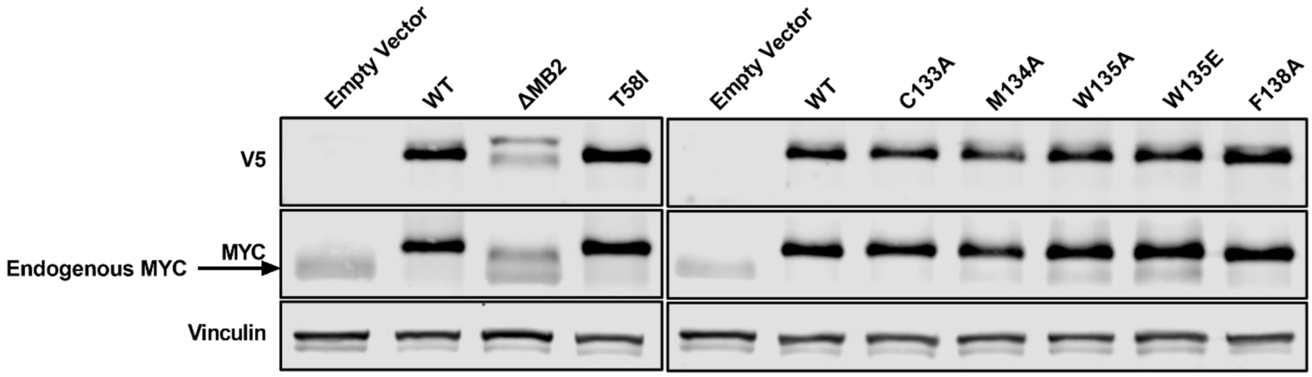

Supplementary Figure 3: MCF10A cells maintain MYC expression with MB2 substitutions. Western blot analysis of each of the MCF10A cell lines created using pCDH-V5-MYC. MB2 Substitutions do not seem to alter ectopic MYC expression levels drastically.
